# Inferring the nature of linguistic computations in the brain

**DOI:** 10.1371/journal.pcbi.1010269

**Published:** 2022-07-28

**Authors:** Sanne Ten Oever, Karthikeya Kaushik, Andrea E. Martin

**Affiliations:** 1 Language and Computation in Neural Systems Group, Max Planck Institute for Psycholinguistics, Nijmegen, the Netherlands; 2 Donders Centre for Cognitive Neuroimaging, Radboud University, Nijmegen, the Netherlands; 3 Department of Cognitive Neuroscience, Faculty of Psychology and Neuroscience, Maastricht University, Maastricht, the Netherlands; University College London, UNITED KINGDOM

## Abstract

Sentences contain structure that determines their meaning beyond that of individual words. An influential study by Ding and colleagues (2016) used frequency tagging of phrases and sentences to show that the human brain is sensitive to structure by finding peaks of neural power at the rate at which structures were presented. Since then, there has been a rich debate on how to best explain this pattern of results with profound impact on the language sciences. Models that use hierarchical structure building, as well as models based on associative sequence processing, can predict the neural response, creating an inferential impasse as to which class of models explains the nature of the linguistic computations reflected in the neural readout. In the current manuscript, we discuss pitfalls and common fallacies seen in the conclusions drawn in the literature illustrated by various simulations. We conclude that inferring the neural operations of sentence processing based on these neural data, and any like it, alone, is insufficient. We discuss how to best evaluate models and how to approach the modeling of neural readouts to sentence processing in a manner that remains faithful to cognitive, neural, and linguistic principles.

## Introduction

Language is not contained in the physicality of speech, sign, or text; rather, the brain must construct meaning from the sensation of the physical input based on internal knowledge. To infer sentence meaning, we need to understand individual words as well as how these words structurally relate to each other. Ding and colleagues [1] showed that the human brain is sensitive to linguistic structure by presenting adjective-noun-verb-noun sentences containing a noun and a verb phrase (NP and VP, respectively). The stimuli are presented at a 4-Hz rate and contain only a 4-Hz acoustic signal. Nonetheless, they find that participants’ MEG responses show peaks not only at the acoustic (4-Hz) rate, but also at the sentence (1-Hz) and phrasal (2-Hz) rate. There has been a wide debate, based on this finding, about what the computational neural principles governing linguistic structure processing are.

One way to explain the Ding and colleagues data [1] is to assume that the brain has explicit models of linguistic structures that are imposed on the stimulus input ([2]; also see [3]). The 1-Hz patterns follow as the brain has an explicit response to sentences. Alternatively, a response to sentence structure is extracted from the statistics in the stimulus input. Frank and Yang [4] showed that by concatenating distributed semantic representations of words, a 1-2-4-Hz pattern also emerges. As such, the authors state that the 1-Hz response does not require an explicit model of sentence structure and distributed associative representations in the brain can explain the data [4].

These two accounts create an interesting dichotomy that is unresolved. Which account provides a “better” mechanistic explanation of what the brain does? Here, we discuss some limitations on the interpretation of models of neural readouts related to linguistic structures. While the debate is open, it is important to shed light on how we should treat modeling of neural data in order to refrain from making unlicensed conclusions. This paper aims to serve as a didactic tool to improve future argumentation on what counts as evidence for hierarchical linguistic structure in neural readouts, and the resulting inferred computations. We have therefore structured our paper by posing (in our opinion) strong statements from each polar view that appear implicitly as well as explicitly in the literature.

## Examples of unfounded inference

### The output of artificial neural nets shows the 1-2-4 Hz without explicitly processing sentences, thus the brain does not explicitly process sentences

Artificial neural networks (ANNs) of different architectures feature a collection of interconnected units that are trained according to a variety of procedures in order to extract statistical patterns in data. They can be trained to predict upcoming words, or can be used to create distributional semantic representations such as word2vec. Frank and Yang [4] showed that a 1-2-4-Hz pattern for sentences can be created by using word2vec representations and extracting the frequency content of the individual dimensions of this vector representation in the sentence. From this, the authors infer that it is not necessary for the brain to form an explicit representation of sentences when it creates the pattern found in the Ding and colleagues [1] data. Instead, they argue that because adjective-noun-verb-noun patterns follow each other, words can be processed sequentially, and the brain could simply be sensitive to the regularities on the word level, instead of creating an integrated representation of the sentence structure. Importantly, these results should not be interpreted as a proof that the brain does not form an abstract sentence representation (and Frank and Yang [4] also do not do this, but discourse in the field has interpreted this finding in this vein). Simply because one model can create the pattern by not using sentence representations does not imply that the brain does not reach the pattern in this way (i.e., the multiple realizability problem, see [5–9]). Indeed, Martin and Doumas [2] have shown that the 1-2-4-Hz pattern can also be created by a model that does encode sentence-like propositions by explicitly representing relationships between words and phrases in time [10]. Based on these two modeling approaches, and the Ding and colleagues [1] data alone, it is impossible via inductive inference to know whether the readout arise from the brain constructing explicit sentences representations or not.

### Artificial neural networks by definition do not explicitly process sentence structure

**Fig 1:**
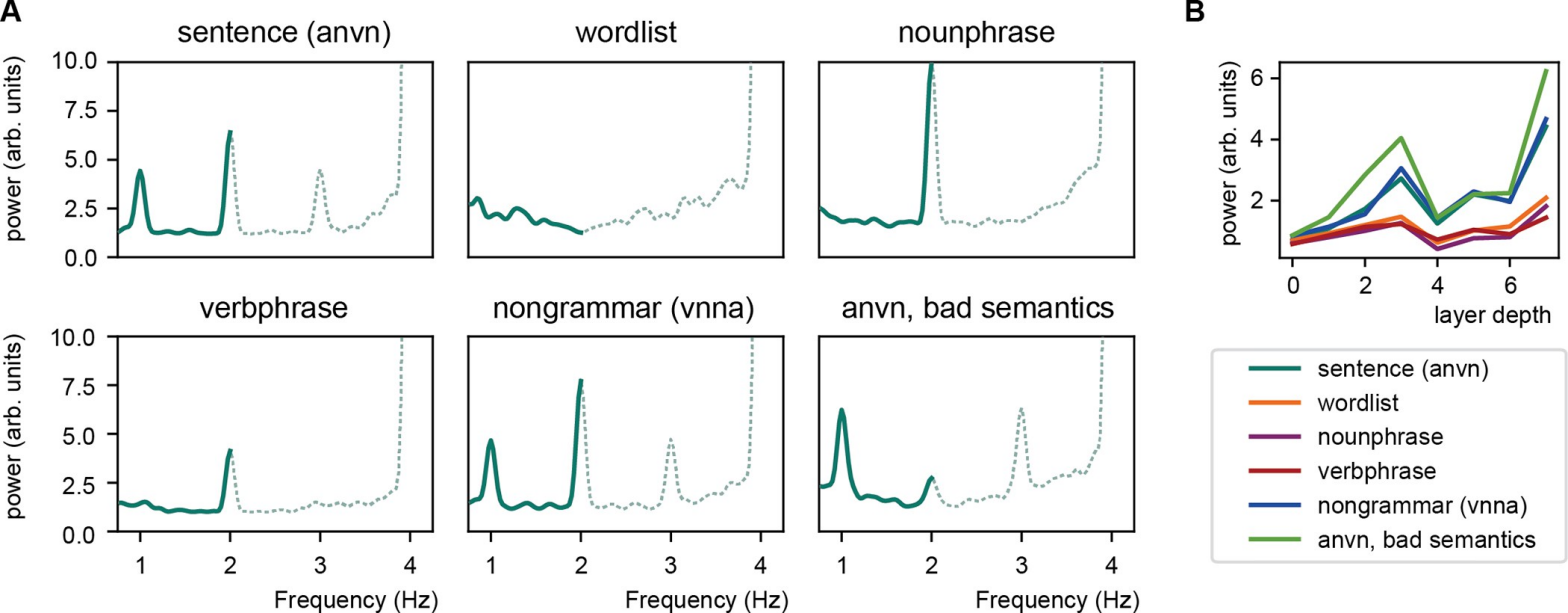
Output of the Berkeley parser. (A) Spectra of the output of the parser using different versions of the Ding and colleagues stimuli (a = adjective, n = noun, v = verb). Dashed lines indicate output from the FFT when interpolating the data by inserting zeros after every word (up-sampling the data from 4 to 8 Hz). (B) 1-Hz response across the different layers of the parser model.

Just as the multiple realizability problem exists across different classes of computational models, it also exists within a single class of models such as ANNs. Indeed, ANNs processing language can achieve a 1-2-4-Hz pattern under vastly different architectures and underlying model goals. Some of these models process sentence structure explicitly, but others do not. As show above, word2vec representations can create a 1-2-4-Hz pattern without directly modeling abstract sentence representations [4]. However, one can also create an ANN whose purpose is to annotate sentences with their linguistic constituents. As these models output how different syntactic structures organize a text, they definitionally contain explicit representations of sentences. One such model is the Berkeley Neural Network Parser, which is trained to parse sentences in syntactic units [11,12]. We investigated how the Berkeley parser behaves using the Ding and colleagues [1] stimuli (Fig 1; for details see https://github.com/sannetenoever/2022_SimLinguisticInferences). What is evident is that just like in [4] or [2], the Berkeley parser also peaks at the sentence rate (Fig 1A). It does so for sentences, but not for word lists, or noun and verb phrases. Interestingly, the model also shows a peak when presented with ungrammatical sentences with uninterpretable semantics, but in which syntactic structure is preserved (viz., shuffling the words within the original sentences; also see [13]). What can also be appreciated is that sentence representations are strongest at the deepest, most abstract layer (Fig 1B). This is expected, as the ultimate goal of the parser is to explicitly represent the sentences at the highest hierarchical level. Since sentence input in the Ding and colleagues data occurs at 1-Hz, the output should contain the 1-Hz representation. While many commonly known models are not explicitly provided with syntactic instruction, viz., word2vec, it cannot be assumed that all ANN architectures definitionally are, as that depends on the input and instructions to the model: Parsers explicitly leverage a representation of syntax. What is more, the above simulation shows that both models—either explicitly instructed to parse the sentence or instructed with a non-grammatical next-word prediction goal—can show the 1-2-4-Hz pattern.

### The output shows a 1-2-4-Hz pattern, so the computation also has to

**Fig 2:**
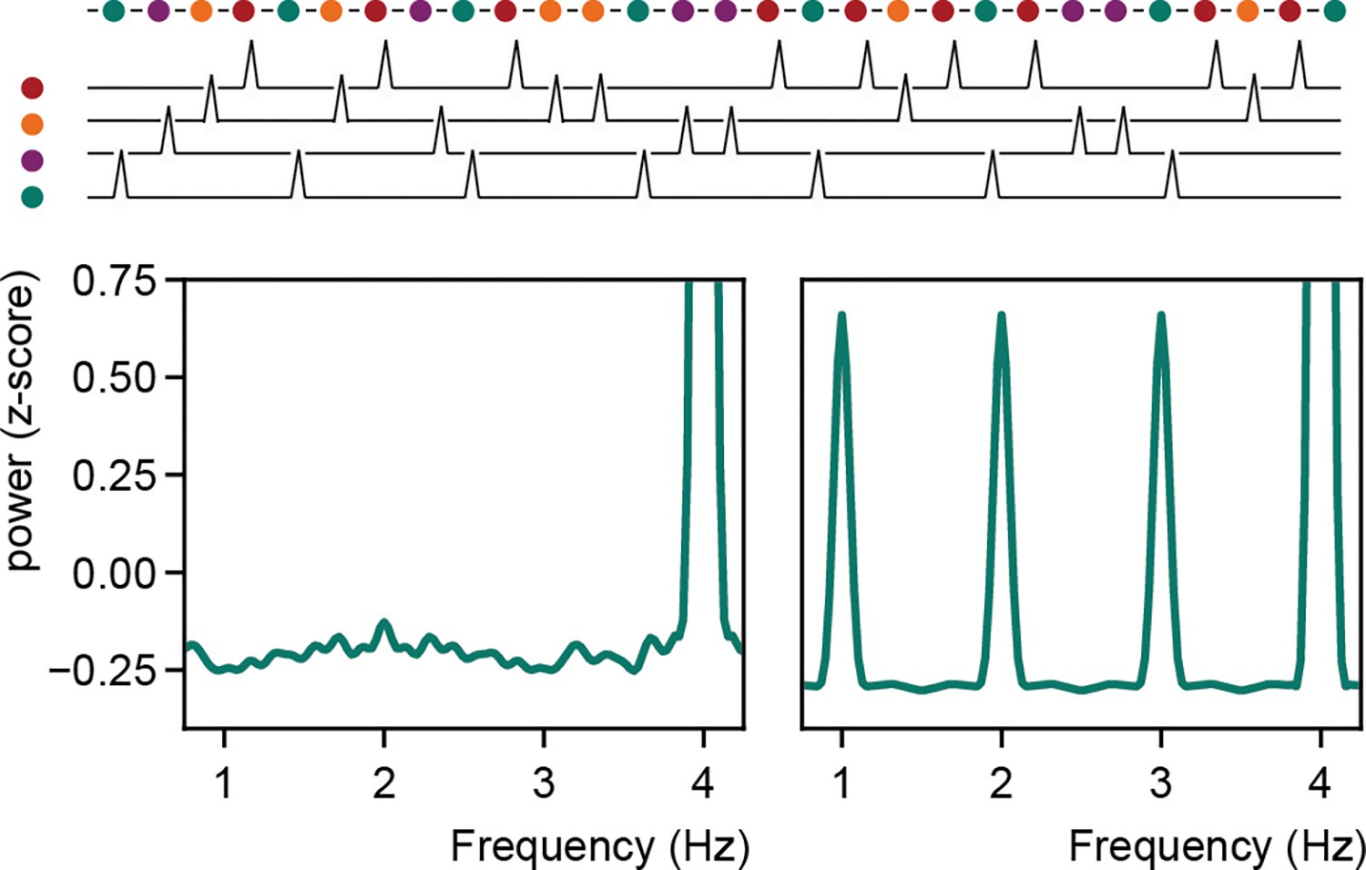
SSVEP studies do not assume any integration of responses. When multiple category stimuli (here different colors) are presented with all a stereotypical response, a low frequency response will occur if any of them is presented at a specific rate (here green). Left: all items randomly presented. Right: single item is presented at a 1-Hz rate.

The 1-Hz peak in the Ding and colleagues [1] paper could imply that there is some integrative process happening that is slower than the individually presented words [14–17]. This interpretation is appealing as we have the inherent feeling that we integrate sequential words into a hierarchical representation that is the sentence, and we know that we arrive at integrated sentence meanings dictated by syntactic structure. However, nothing about the neural data shows that this integration necessarily has to occur. Indeed, in similar visual steady-state evoked response studies random pictures are presented and every *n*th picture represents some specific class like faces [18–20]. If one is sensitive to the class, one finds a peak at the facial rate (Fig 2). One finds the peak as every *n*th stimulus something different happens. The computation does not necessarily happen at this rate, but the peak occurs at this rate as some event happens at that rate. Such a pattern has been shown for faces [18], emotions [21], single words [22], or arbitrarily created changes transitional probabilities [23]. That it is not necessary that the computation happens at the extracted rate is what Frank and Yang [4] argue in the case of sentences, and is also what happens for the output of the Berkeley parser when using ungrammatical sentences (Fig 1). In sum, one has to be cautious in interpreting effects at slow rates as an integrative process when a process simply repeating itself at a slower rate could generate the effect.

### Predictive value is always the ultimate goal in cognitive and neural science

Modern ANN methods have a strong focus on predicting data as best as possible. Intuitively, this seems a good starting point for scientific practice; however, there are serious issues with putting predictive value on a pedestal. While the goal for many ANN methods is to predict new data, in the cognitive and neural sciences, the goal is to explain the capacities of a system and to understand the mechanisms that lead to observed data. The only way to do this is to pose hypotheses about brain mechanisms, constrained through abductive inference based on what we know to an acceptable degree of verisimilitude about language and the brain, and test explicitly for these mechanisms [7,8,15,24]. Some forms of data science applications simply replace the thing we aim to understand (e.g., the brain, behavior, the capacity of a system for human language) with another black box (e.g., an ANN model; [25,26]). A simple classical cognitive box model can add to the level of understanding of brain operations, even if it does not do a great job at quantitively predicting the data. To use an ANN to explain brain mechanisms, at a minimum one must show that the operations and parameters of a complex model are related to brain operations [27–29]. This last step is often difficult. Thus, it often is still very valuable to work with models that provide explicit claims about the features that influence the model (e.g., encoding models such as regression).

### When a model is trained on more data it is more realistic

It is easy to be impressed by large language models that are trained on an incredible amount of data and have high predictive power. As cognitive scientists and neuroscientists, our aim is to explain brain computation, and we therefore must stay true to the data that the brain processes. It has often been argued that the brain cannot be like an ANN because it does not have access to so much training data [26,30]. Sometimes, learning a simple rule can account for some transferable skills the brain possesses, while contemporary language models and deep nets more broadly, with more data, training, energy use, and compute, have a harder time with task transfer [31–33]. However, this state of affairs does not exclude that for other tasks, the brain might employ a strong associative approach. When interpreting computational models, one must determine whether the brain realistically had access to, and has the capacity for processing the data put into a computational model to obtain a desired result. If the required data size and computational power are super- or inhuman, rule learning may be a form of computational compression that is more explanatory in the context of cognitive modeling.

### Statistical models are “simpler” or “more parsimonious” than hierarchical models

Frank and Yang [4] argue that representing the Ding and colleagues [1] data with only distributed lexical representations as in word2vec, compared to hierarchal representations [2]—which need both lexical and syntactic representations—is more parsimonious. However, word2vec is created by using a huge corpus of data with thousands of connections between many layers to squeeze the distributional information into a vector. Thus, even if word2vec by itself could be parsimonious, creating it is clearly not. Moreover, a word2vec representation is far from purely lexical as distributional patterns latently contain indirect syntactic information. Word2vec is created by forming associations with all possible neighboring words. Therefore, during the creation of a word2vec representation, the model uses sentence or at least neighboring word information (although not necessarily in a hierarchical manner). While indeed, creating the Ding and colleagues [1] pattern in neural readout cannot be said to require building a syntactic structure during sentence processing, word2vec can create the 1-Hz pattern precisely because it puts syntactic classes in neighboring positions in its vector representation. In any case, if one assumes the brain has a word2vec representation, it needed to construct it by integrating information across the sentence like the ANN does when creating the word2vec.

## How can we conclude anything

It is difficult to conclude whether statistical or hierarchical models can explain better the Ding and colleagues [1] data as both model classes can be made to recreate and predict the neural readout. Even though it is difficult to know whether a computational implementation is the one the brain uses [34], computational modeling still provides a means to be explicit about cognitive and neural processes at hand and thereby improve theory building [7]. The field will progress, but only if the outcomes of models and empirical data are interpreted, compared, and synthesized in a careful manner.

The starting point of every model should always be the explanandum: the process or capacity of a system that we aim to explain [24,35,36]. For cognitive neuroscientists, this is the brain operation that is, or that leads to or comprises, a cognitive operation. In trying to model sentence processing, deriving a processing mechanism that yields compositionality (viz., compositionality entails that the meaning of sentences is determined by that of individuals words and the rules used to combine them [6,37]) seems absolutely crucial (or, alternatively providing a reason why compositionality is not necessary for sentence processing) for a theory of language representation and processing. The structured meaning that we experience from sentences is also the explanandum of cognitive models of language; if a model does not provide functionally equivalent “linguistic” output, then it does not account for the phenomenon, nor the capacity set out to be modeled. If one ignores these core principles of sentence processing, but goes directly toward trying to explain the neural data without context for abduction, then the model does not stay true to its epistemic goal.

If our goal is to explain neural computation, then similarly as above, only models that stay true to possible neurophysiological implementations of neural computation can ultimately be valid. One needs to have reasonable assumptions about how neuronal populations or ensembles could perform the computation; this should be explicitly stated, argued, and considered. If one models sentence processing as a hierarchical linguistic process, it is a reasonable starting point to investigate hierarchical, temporal [2,38,39], and anatomical organization in brain computation [40,41]. If one models sentence processing as a sequential process, one has to be explicit about the sequential steps taken in the brain. But one cannot simply assume that because a model fits the data, the brain computation is equivalent to what is instantiated in the model [8].

In an ideal world, a model of sentence processing should be able to explain not only the frequency-tagging data as generated by Ding and colleagues [1], but also stay faithful to the vast psycholinguistic and event-related brain potential (ERP) literature on how sentences are processed differently from word lists or unstructured acoustic input [42–44]. From this literature, we know that the brain is sensitive to the semantic and syntactic properties of sentences and discourses, indicating that the brain integrates words into phrases and sentences (and thus, does not treat words as independent units). This literature makes it unlikely that the only thing the brain does is create a word-by-word word2vec-like representation [4], but instead words need to be integrated (either sequentially or hierarchically). Putting too much emphasis on a single experimental finding can result in epistemic myopia; a simple way to safeguard against this is to require that theoretical and neurocomputational models explain a wide range of findings. More importantly, the ultimate goal of a cognitive model of language representation and processing is to explain the human capacity for natural language and language behavior. While experiments provide tailored designs that can discriminate between competing models, they are often intentionally far from natural circumstances in order to better orthogonalize factors. Exclusive focus on stimuli that are never encountered in daily life (such as in many experimental studies) and only become relevant in a lab setting can often obscure the original goal of the model, which is to explain a cognitive process or capacity, not only a specific dataset. It is therefore necessary that experimental approaches are complemented with studies using naturalistic paradigms [45,46] that more directly investigate the operations of interest, namely naturalistic spoken language comprehension.

Ultimately, every new discovery such as the Ding and colleagues [1] study leads to new questions. Models should be updated when new experimental data that can differentiate between proposed accounts (see, e.g., [13,47,48]) becomes available. Similarly, models must be explicit about the operations that hierarchical or sequential syntactic structure building draws upon and how they might operate in the brain in order to create predictions that can be experimentally tested [15,39]. Only through systematic (theoretical) modeling one can generate model predictions which then can be experimentally tested, though prediction alone is not sufficient to explain how and why a behavior, capacity, or phenomenon is the way it is. Careful modeling can be followed by new experimental paradigms that put both principles and predictions to the test, which may lead to different types of evidence than the Ding and colleagues [1] study can provide by itself (see e.g., [45,46]). This type of data-to-model-to-data cycle catalyzes theoretical models, as well as theories of brain computation, and can disentangle different accounts of sentence processing [7,49]. For example, some studies have fit predictions from different computational models onto electroencephalographic (EEG) and functional magnetic resonance imaging (fMRI) data and have shown a better fit for hierarchical rather than sequential models for natural sentence processing [50,51]. These studies provide a first means to push the debate forward and highlight the value of comparing different computational models with each other to advance our understanding of the brain.

In sum, we believe that computational modeling is vital for understanding sentence processing through the lens of neural readouts (e.g., frequency-tagging, mutual information, phase synchronization, and connectivity signals). In order to be explanatory, models must compute linguistically-sufficient representations via explicit cognitive operations that are specified within a system architecture that stays faithful to neurophysiological principles. Furthermore, we should not be bewitched by the predictive power of our models, but instead should judge whether the proposed model is a likely model of brain function, and whether it explains more than just a single dataset. Only by generating explicit models we can differentiate, and ultimately integrate, sequential and hierarchical accounts, as well as arrive at modeling the neurocomputational principles on which these processes operate. There is no doubt that we need more modeling, as well as experimental, theoretical, and formal work, in order to reach these goals.
